# The matrix protein of rabies virus binds to RelAp43 to modulate NF-κB-dependent gene expression related to innate immunity

**DOI:** 10.1038/srep39420

**Published:** 2016-12-21

**Authors:** Youcef Ben Khalifa, Sophie Luco, Benoit Besson, Florian Sonthonnax, Medhi Archambaud, Jonathan M. Grimes, Florence Larrous, Hervé Bourhy

**Affiliations:** 1 Institut Pasteur, Unité Dynamique des Lyssavirus et Adaptation à l’Hôte, 25 rue du docteur Roux, 75015 Paris, France; 2 Université Paris Diderot, Sorbonne Paris Cité, Cellule Pasteur, rue du docteur Roux, 75015 Paris, France; 3 Division of Structural Biology, Wellcome Trust Centre for Human Genetics, University of Oxford, Oxford, United Kingdom; 4 Diamond Light Source Limited, Harwell Science and Innovation Campus, Didcot, OX11 0DE, UK

## Abstract

The matrix (M) protein of wild isolates of rabies virus such as Tha (M-Tha) was previously shown to be able to interact with RelAp43, a protein of the NF-κB family, and to efficiently suppress NF-κB-dependent reporter gene expression, in contrast with the vaccine strain SAD. Here, we analyze the mechanisms involved in RelAp43-M protein interaction. We demonstrate that the central part of M-Tha, and the specific C-terminal region of RelAp43 are required for this interaction. Four differences in the corresponding amino acid sequences of the M-Tha and M-SAD are shown to be crucial for RelAp43 interaction and subsequent modulation of innate immune response. Furthermore, the capacity of M-Tha to interact with RelAp43 was shown to be crucial for the control of the expression of four genes (*IFN, TNF, IL8* and *CXCL2*) during viral infection. These findings reveal that RelAp43 is a potent regulator of transcription of genes involved in innate immune response during rabies virus infection and that the M protein of wild isolates of rabies virus is a viral immune-modulatory factor playing an important role in this RelAp43-mediated host innate immunity response in contrast to M protein of vaccine strains, which have lost this property.

The innate immune response to virus infection involves activation of pattern recognition receptors (PRR) and transcriptional induction of many pro-inflammatory cytokines, including type I interferons (IFNs). This activation of gene expression is controlled by the activities of some regulatory factors, such as interferon-regulatory factors 3 and 7 (IRF-3 and -7 respectively), activator protein 1 (AP1) and a family of proteins called nuclear factor of the kappa light chain enhancer of B cells (NF-κB). Until recently, the NF-κB pathway was described as a family of five proteins in mammalian cells: p65/RelA, p50 (NF-κB 1), p52 (NF-κB 2), c-Rel and RelB. This list was recently enlarged with the characterization of a splicing variant of p65/RelA, called RelAp43^1^. All NF-κB proteins share a structurally conserved N-terminal region, the Rel Homology Domain (RHD), which is critical for homo- or hetero-dimerization, nuclear localization, binding to DNA on κB sites and interaction with inhibitory proteins IκB. p50 and p52 form transcriptionally active heterodimers with either p65/RelA, c-Rel or RelB^2^,^3^. RelAp43 can also form heterodimers with the five other members of the NF-κB family, including p65/RelA and p50, and activate transcription of NF-κB dependent genes^1^. Rel proteins (p65/RelA, c-Rel and RelB) contain a C-terminal transactivation domain (TAD), which is lacking in RelAp43, p50 and p52^1^. Therefore, p50 and p52 form homodimers with no intrinsic ability to activate transcription and transcriptionally active heterodimers in association with p65, cRel, and RelB^4^.

In the majority of cell types, NF-κB is kept inactive in the cytoplasm through association with an inhibitory protein of the IκB family, which includes IκBα, IκBβ, IκBε, p105 and p100 (the cytoplasmic precursors of p50 and p52 respectively). Most of the signals that lead to activation of NF-κB, such as cytokines, various stress signals, and viral or bacterial infections, activate a high molecular weight complex containing a serine-specific IκB kinase (IKK). IKK is largely composed of three distinct subunits: the two related catalytic kinases-IKKα and IKKβ, and NEMO^5^. Activated IKK then phosphorylates IκB on specific residues, which is the signal for ubiquitination and proteasomal degradation of the inhibitor. As a consequence, free NF-κB dimers enter the nucleus and activate transcription of their target genes by binding κB sites in the promoter region of numerous genes^6^. Since the dimers have different transcriptional activity at each promoter, dimer exchange allows fine tuning of the NF-κB response^4^.

RNA viruses like the *Rhabdoviridae*, and in particular those belonging to the *Lyssavirus* genus, have developed multiple and synergistic strategies to counteract the innate immunity^7^,^8^. Numerous recent studies have thrown further light on how these viruses counteract IRF3/7-dependant IFN induction and IFN signaling^9^,^10^,^11^,^12^,^13^,^14^,^15^,^16^,^17^. However, their potential to interfere with NF-κB has been less intensively studied, although transcriptional activation of the IFN-β gene is known to require assembly of an enhanceosome containing IRF3/7 but also other transcriptional factors such as ATF-2/c-Jun and NF-κB^2^,^18^,^19^. Enhanceosome assembly occurs only after viral infection and not in response to other signals that can separately activate each of the transcription factors^20^,^21^. This combinatorial mechanism is based on the fact that virus infection is the only known signal that can activate all of the IFN-β transcriptional activators simultaneously^22^,^23^.

The M protein of lyssavirus, the agent of rabies, is a small protein (~20–25 kDa), forming oligomers that bind to the outside of the nucleocapsid, giving rigidity to the virion structure^24^,^25^. Beside its structural role in the virion of lyssavirus, the M protein is a potent modulator of apoptosis after lyssavirus infection^26^,^27^,^28^. It is also able to target RelAp43, thus inducing an inhibition of NF-κB signaling and a reduction in IFN-β transcription^1^. Furthermore, this modulation is highly dependent on the strain of lyssavirus considered. The ability of the M protein of lyssavirus to interact with RelAp43 and to inhibit the induction of IFN-β, providing a means to evade the anti-viral innate immunity, is lost in vaccinal strains^1^.

In this study, we characterize the binding site of the M protein of lyssavirus on RelAp43. We show that the central part of the M protein encompassing two α helices and a β strand (amino acids 67 to 110) and the C-terminal region of RelAp43 are required for this interaction. In this segment of the M protein, amino acids in positions 77, 100, 104 and 110 are critical for its interaction with RelAp43 and its inhibitory effect on NF-κB signaling. We demonstrate that the inhibitory effect of M protein of wild isolates of lyssavirus on NF-κB signaling is mediated by its action on RelAp43. Although lacking the TAD, we showed that RelAp43 is able to modulate cellular genes involved in innate immunity and NF-κB signalling and we confirmed *in vivo* that Tha virus hijack RelAp43 signaling to control the induction of the TNF. These findings identify RelAp43 as a target of choice for viral interference and the lyssavirus M protein appears as a potent viral immune-modulatory factor that prevents NF-κB genes expression.

## Results

### The specific C-terminal region of RelAp43 is targeted by the amino acids 67 to 110 of M-Tha

**Figure 1:**
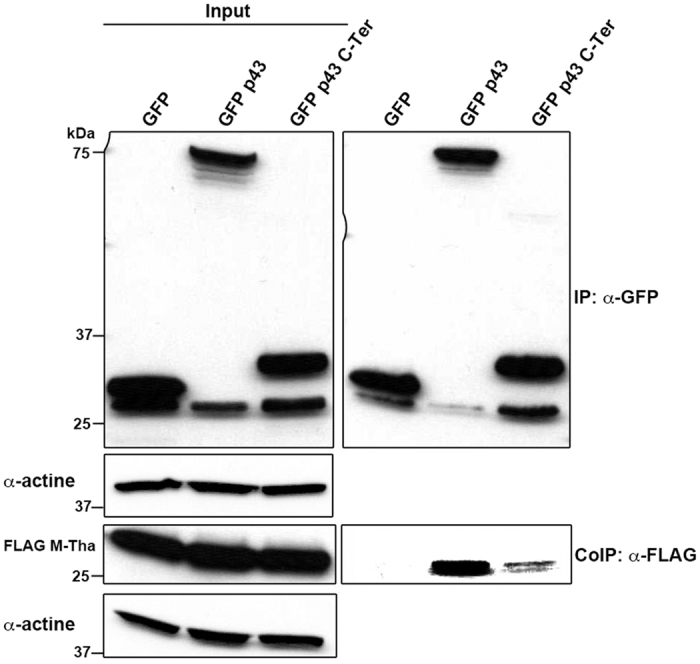
M-Tha interacts with the specific C-terminal region of RelAp43. HeLa cells were co-transfected with FLAG-tagged M-Tha and GFP as a negative control, GFP p43 as a positive control or GFP p43 C-Ter. Co-IP were performed using GFP-Trap and the presence of GFP or FLAG-tagged protein was analyzed by western blot using specific antibodies in cell lysates either before (Input, left panel) or after IP (right panel). The western blots depicted here are the results of a representative experiment (three repeats).

In a previous study, we showed that M-Tha was specificaly interacting with RelAp43 while neither RelA nor the conserved RHD of both proteins could interact with M-Tha^1^. In order to identify the RelAp43 region that interacts with M-Tha, we co-transfected vectors expressing FLAG M-Tha with vectors expressing GFP as a negative control, GFP p43 as a positive control and GFP p43 C-Ter (with the 33 amino acids specific of RelAp43). As expected, co-immunoprecipitation (co-IP) experiments showed that GFP p43 C-Ter is also able to interact with FLAG M-Tha, although less efficiently than the full length GFP p43 (Fig. 1). Thus, the short specific sequence of 33 amino acids of RelAp43 seems to be involved in the interaction with M-Tha.

**Figure 2:**
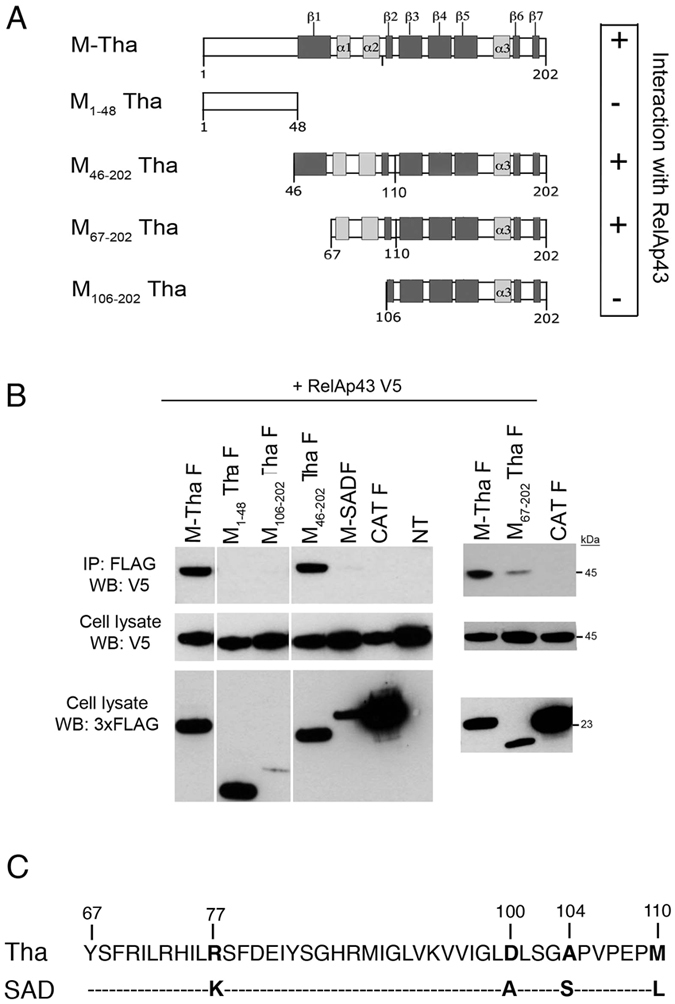
Amino acids 67 to 110 of M-Tha are involved in the interaction with RelAp43. (**A**) Schematic representation of the truncated mutants of M-Tha used in this study. Their capacity of interaction with RelAp43 is summarized on the right part of the Figure (+: interaction with RelAp43; - : absence of interaction). (**B**) HeLa cells were used to co-express V5-tagged RelAp43 in combination with the FLAG-tagged CAT as a negative control or the indicated FLAG-tagged M construct. After 24 h, cells were lysed, protein expression in cell lysates was controlled (cell lysate: WB V5; cell lysate: WB 3xFLAG) and co-IP experiments were performed using anti-FLAG M2 beads. Interaction of RelAp43 with the different M constructs or CAT was visualized by western blot (IP: 3xFLAG; WB: V5). The western blots depicted here are the results of a representative experiment (three repeats). (**C**) Alignment of the 67 to 110 amino acids sequences of M-Tha and M-SAD using the ClustalX software. Four differences appeared in the sequences (in bold), respectively at position 77 (R in M-Tha, K in M-SAD), 100 (D in M-Tha, A in M-SAD), 104 (A in M-Tha, S in M-SAD) and 110 (M in M-Tha, L in M-SAD).

To map the interacting region on M-Tha protein, several truncated mutants were produced: M_1–48,_ M_46–202_, M_67–202_ and M_106–202_ (Fig. 2A). These overlapping mutants were designed to respect motifs of secondary structures of the M protein as described for the related Lagos bat virus^24^ and were tested for their ability to interact with RelAp43^1^. To this aim, we co-transfected plasmids coding for FLAG-tagged M-Tha (FLAG M-Tha) and truncated mutants with a plasmid coding for V5-tagged RelAp43. As controls, we transfected also Flag-tagged CAT and FLAG-tagged M-SAD, the matrix protein of a vaccine strain of lyssaviruses not able to interact with RelAp43^1^. As expected, CAT and M-SAD proteins did not interact with RelAp43. Again, co-IP experiments were performed to study the interaction between the different M constructs and RelAp43 (Fig. 2B). As expected, CAT and M-SAD proteins did not interact with RelAp43. The truncated mutants M_1–48_ and M_106–202_ were not able to interact with RelAp43, meaning that the unstructured N-terminal region and the second half of M-Tha are not necessary for the interaction. Moreover, while the M_46–202_ shows a stronger co-IP of RelAp43 than M_67–202_, the latter is enough to interact with RelAp43. Therefore, the 67–110 region was considered as the minimal region necessary for M-Tha/RelAp43 interaction.

Then, we used the Clustal X software to perform an alignment focused on the 67 to 110 amino acid sequences of M-SAD and M-Tha (Fig. 2C). On this fragment, M-Tha and M-SAD differ only by 4 amino acids: R77K, D100A, A104S and M110L (M-Tha to M-SAD).

### Residues 77 and 104 are necessary but not sufficient for the inhibition of NF-κB signaling mediated by M-Tha

To investigate whether the mutation of one or more of these positions in M-Tha could account for the loss of interaction with RelAp43, we constructed single to quadruple mutants of M-Tha by substituting M-Tha residues 77, 100, 104 or 110 by those observed in M-SAD sequence (Fig. 2C).

**Figure 3:**
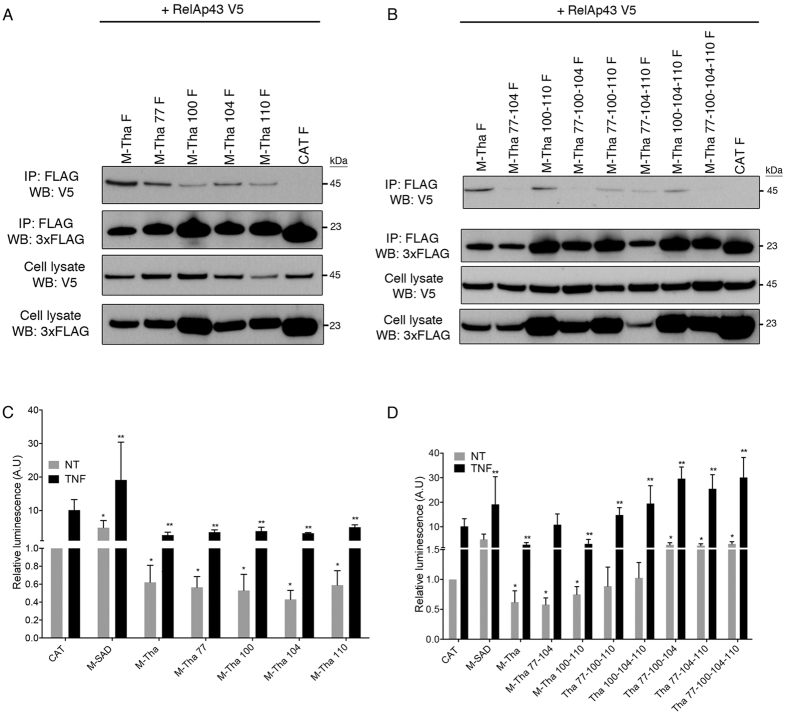
Residues at positions 77 and 104 are necessary for the interaction of M-Tha with RelAp43 and should be coupled with mutations D100A and/or M110L to restore a level of activation of the NF-κB reporter vector comparable to M-SAD. (**A** and **B**) HeLa cells were used to co-express V5-tagged RelAp43 in combination with the indicated FLAG-tagged single (**A**) or multiple (**B**) mutants of M-Tha. After 24 h, cells were lysed, protein expression in cell lysates was controlled (cell lysate: WB V5; cell lysate: WB 3xFLAG on the Figure) and co-IP experiments were performed using anti-FLAG M2 beads. Interaction of RelAp43 with the different M constructs or CAT was visualized by western blot (IP: 3xFLAG; WB: V5 on the picture). The western blots depicted here are the results of a representative experiment (three repeats). (**C**) Modulation of NF-κB activation in 293T cells in the presence of single or double mutants of M-Tha compared to M-Tha, M-SAD or CAT. The NF-κB pathway was exogenously activated using 10 ng/mL TNF-α during 5 h (black bars) or left untreated (grey bars). The CAT control without the TNF treatment was used as the reference condition. (**D**) Modulation of NF-κB activation in 293T cells in the presence of triple or quadruple mutants of M-Tha compared to M-Tha, M-SAD or CAT. The NF-κB pathway was exogenously activated using 10 ng/mL TNF-α during 5 h (black bars) or left untreated (grey bars). The CAT control without the TNF treatment was used as the reference condition. Significant results (p < 0.05) in comparison to untreated (*) and TNF treated (**) CAT transfected cells were annotated.

First, the capacity of interaction between V5-tagged RelAp43 and each of the different FLAG-tagged M-Tha constructs or a FLAG-tagged CAT control was studied by co-IP experiments. All four single mutants of M-Tha: M-Tha 77, M-Tha 100, M-Tha 104 and M-Tha 110 retained the ability to interact with RelAp43 although less efficiently than wild type M-Tha (Fig. 3A). Interestingly, double mutant M-Tha 100–110 kept a relative ability to interact with RelAp43 conversely to M-Tha 77–104. As for the multiple mutants of M-Tha, the interaction with RelAp43 was considerably reduced in the case of M-Tha 77-100-110 and M-Tha 100-104-110 and almost totally lost with M-Tha 77-100-104, M-Tha 77-104-110 and M-Tha 77-100-104-110 (Fig. 3B). Thus, all these results attested that the binding of M-Tha to RelAp43 is largely dependent on the residues 77 and 104.

Then, we studied the capacity of the various truncated mutants of M to modulate the NF-κB pathway following treatment with the NF-κB inducer TNF-α for 5 h, since there is a strong correlation between the ability of the M protein of lyssaviruses to interact with RelAp43 and its inhibition effect on the NF-κB pathway^1^. As expected, the transfection of M-Tha with or without addition of TNF induced an inhibition of the luciferase reporter gene under control of κB sites in contrast to the control CAT (Fig. 3C). On the contrary, M-SAD transfected cells showed an activation of the NF-κB signaling in contrast to the control CAT (Fig. 3C). We then analysed the effect of single, double and triple mutants of M-Tha on the NF-κB signaling. Three categories of modulations were reported. The four single mutants of M-Tha (Fig. 3C) and the double mutants M-Tha 100-110 and M-Tha 77–104 (Fig. 3D) exhibited an inhibition of the NF-κB reporter compared to the control CAT, either with or without any TNF treatment (excepted M-Tha 77–104 after TNF stimulation which reach the CAT control level). Two of the triple mutants, M-Tha 77-100-110 and M-Tha 100-104-110 (Fig. 3D) showed a level of NF-κB activity similar to that observed with CAT in absence of TNF (p < 0.05) and a level similar to M-SAD in presence of TNF. Conversely, M-Tha 77-100-104, M-Tha 77-104-110 and the quadruple mutant M-Tha 77-100-104-110 exhibited a level of luminescence comparable to that achieved by expression of M-SAD in absence and in presence of TNF (Fig. 3D). This suggests that these triple and quadruple mutants of M-Tha is not able to inhibit the NF-κB signaling and favour its activation similarly to M-SAD (with or without TNF treatment).

Altogether, these results indicate that mutations R77K and A104S although sufficient for the loss of the binding of M-Tha to RelAp43 in co-IP experiments, should be coupled either with D100A or with M110L to fully block inhibition of the NF-κB pathway and reach a level comparable to that observed with M-SAD.

### Mutations of residues 77 and/or 104 of M-SAD are sufficient to restore its interaction with RelAp43 and to inhibit the activation of NF-κB signaling

In order to confirm the importance of the identified residues of M-Tha in the interaction with RelAp43 and the subsequent modulation of the NF-κB pathway, we decided to construct several mutants of M-SAD by replacing residues at single or multiple selected positions with those observed in M-Tha (Fig. 2C).

**Figure 4:**
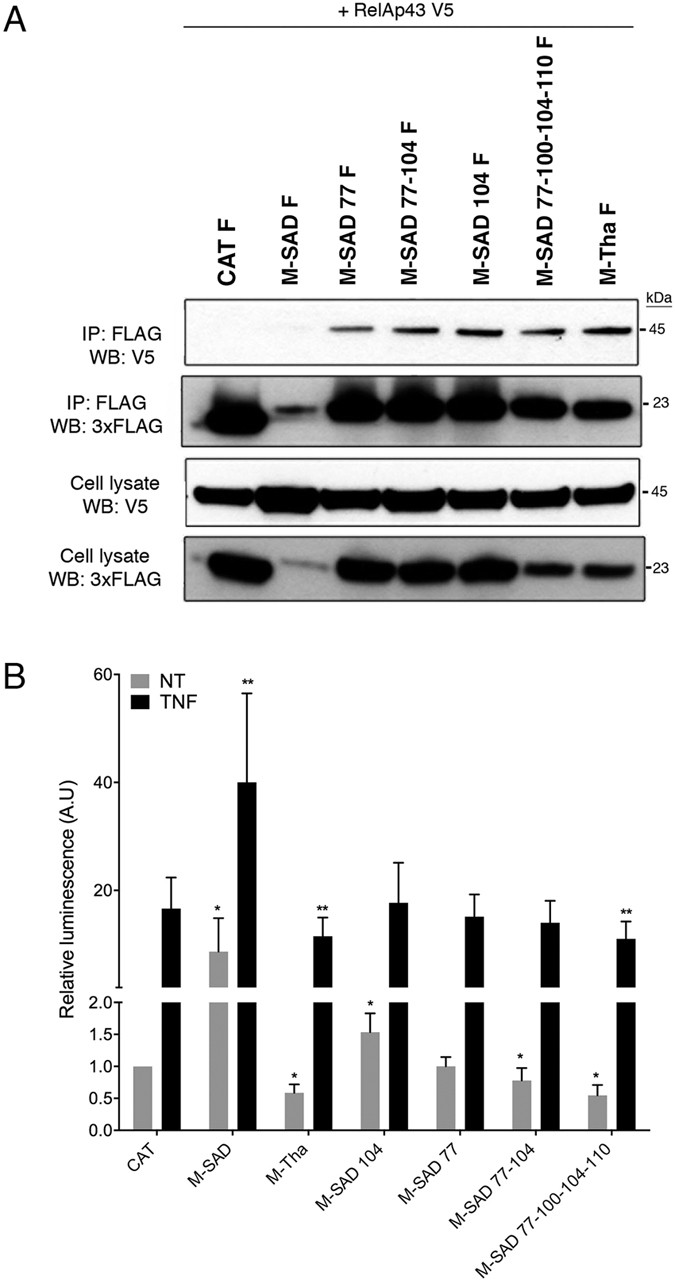
Mutation K77R or S104A in M-SAD is sufficient to induce its interaction with RelAp43 and should be coupled with mutations D100A and M110L to abolish the M-SAD activation of the NF-κB reporter vector. (**A**) HeLa cells were used to co-express V5-tagged RelAp43 in combination with the FLAG-tagged CAT as a negative control, M-Tha as a positive control or the indicated FLAG-tagged single, double or quadruple mutants of M-SAD. After 24 h, cells were lysed, protein expression in cell lysates was controlled (Input: WB V5 and WB 3xFLAG) and co-IP experiments were performed using anti-FLAG M2 beads. Interaction of RelAp43 with the different M constructs or CAT was visualized by western blot (IP: 3xFLAG; WB: V5). The western blots depicted here are the results of a representative experiment (three repeats). (**B**) Modulation of NF-κB activation in 293T cells in the presence of single, double or quadruple mutants of M-SAD compared to M-Tha, M-SAD or CAT. The NF-κB pathway was exogenously activated using 10 ng/mL TNF-α during 5 h (black bars) or left untreated (grey bars). The M protein of vaccine strain SAD with the TNF treatment was arbitrary considered as a reference. Significant results (p < 0.05) in comparison to untreated (*) and TNF treated (**) CAT transfected cells were annotated.

To investigate whether these mutations could also restore the interaction of M-SAD with RelAp43, we performed Co-IP experiments with V5-tagged RelAp43 and each of the different FLAG-tagged M-SAD constructs, a FLAG-tagged CAT negative control or the FLAG-tagged M-Tha positive control. The single and multiple M-SAD mutants were all able to interact with RelAp43 to a similar extent compared to M-Tha (Fig. 4A). Thus, these results indicate that substitutions of M-SAD residues at position 77 and/or 104 by those observed in M-Tha were sufficient for M-SAD to interact with RelAp43.

Then, we used the luciferase reporter vector under the control of κB sites and assessed the modulation by M-SAD mutants of the NF-κB pathway after 5 hours of TNF treatment. As shown above, a significant (p < 0.05) inhibition of the NF-κB reporter vector was observed in the presence of M-Tha in contrast to the CAT control while M-SAD activated the promoter, with or without TNF treatment (Fig. 4B). The single mutant M-SAD 104 induced a significantly higher (p < 0.05) luminescence signal of the NF-κB reporter in absence of TNF compared to CAT although in a less extend than M-SAD. However, the same mutant exhibited an induction of NF-κB reporter in presence of TNF similar to CAT and significantly lower than M-SAD (p < 0.05). Moreover, the effect of M-SAD on the activation of the NF-κB reporter was abolished (and similar to CAT), with or without TNF treatment, when single mutation at residue 77, double mutations 77-104 or quadruple mutations 77-100-104-110 were introduced (Fig. 4B). Further, the quadruple mutant M-SAD 77-100-104-110 was the only one able to reach a level of inhibition of NF-κB signaling similar to M-Tha (40%) compared to the control CAT.

### Tha virus controls host cell response to the infection through the interaction M-RelAp43

After its initial role in the launch of the type I IFN antiviral innate immune response, NF-κB then switches to regulating a distinct subset of non-IFN genes, including those involved in inflammation and cell survival^29^. Additionnaly, previous work from our lab already demonstrated that RelAp43 is able to modulate the expression of some genes involved in antiviral immunity^1^.

Therefore, we quantified the expression level of several genes by real-time PCR in infected HeLa cells and to further demonstrate the capacity of M-Tha to inhibit the NF-κB pathway, we applied or not a treatment with TNF for 5 hours. Based on the results obtained on a human NF-κB signaling target RT^2^ profiler PCR array (Supplementary Table S1), we chose to study several genes for which cells failed to elicit the expression during wild isolate Tha infection versus vaccinal strain SAD (*CXCL2, IFNβ, IL8, TNF*).

As previously described, infection of HeLa cells with SAD induced higher viral titers than with Tha^1^. To investigate if these modulations of gene expression are due to the targeting of RelAp43 by the Tha matrix protein and to avoid differences in viral growth that could interfere with the results, we rescued a Tha recombinant virus mutated on residues 77-100-104-110 of the M protein, named Th4M. The growth of this recombinant virus was studied during 72 hours and the expression of P and M proteins was analysed by western blot (Supplementary Figure S1). Tha and Th4M presented similar levels of P and M protein expression after 72 h of infection despite a slight decrease (half a log) in viral titer in the case of Th4M. These isogenic viruses differing only in the RelAp43 targeting were used in parallel to SAD to fully understand the modulations of gene expression.

**Figure 5:**
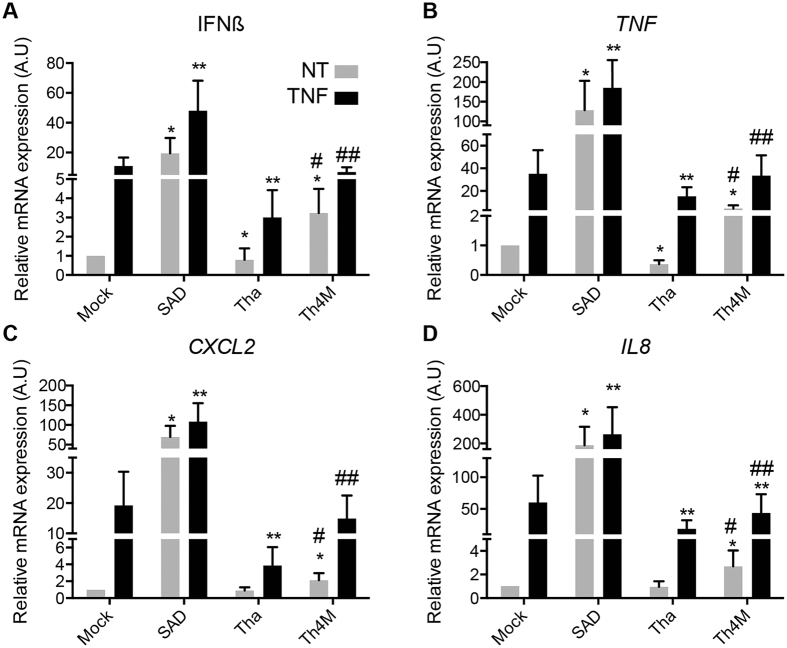
M-Tha positions 77, 100, 104, 110 are important for viral escape. HeLa cells were infected by Tha, Th4M or SAD. After 43 hours of infection, the NF-κB pathway was exogenously activated using 10 ng/mL TNF-α during 5 h (black bars) or left untreated (grey bars) and total RNA was extracted. The expression level of the following genes: *IFNβ* (**A**)*, TNF* (**B**)*, IL8* (**C**)*, CXCL2* (**D**) was studied by RT-qPCR analysis. Significant results (p < 0.05) compared to the untreated (*) or TNF treated (**) mock and to the untreated (^#^) or TNF treated (^##^) Th4M infected cells are annoted. The levels of gene expression were normalized according to the level of *GAPDH* reporter gene in non-infected and untreated cells.

In the mock control, TNF treatment was able to elicit a strong induction of *IFNβ, TNF, CXCL2*, and *IL8* genes (Fig. 5A to D). SAD infection with or without TNF significantly up-regulated (p < 0.05) the expression of all these genes compared to Tha, Th4M and uninfected cells (Fig. 5A–D). In Tha infected cells, *IFNß* and *TNF* genes were significantly (p < 0.05) downregulated, regardless of TNF treatment compared to the mock or Th4M infected cells (Fig. 5A and B). Moreover, the expression of *CXCL2* and *IL8* was the same in Tha infected cells than in mock control without TNF and, but in presence of TNF, their expression was significantly (p < 0.05) inferior to the mock infected cells. Finally, the Th4M induced a significant increase (p < 0.05) of *IFNß, TNF, CXCL2* and *IL8* transcription compared to the control or Tha (Fig. 5A–D), with or without TNF treatment, although with still a lower level than that observed in SAD-infected cells. Conversely, in presence of TNF, Th4M induced a level of transcription comparable to the mock infected cells.

### Tha virus hijacks RelAp43, a NF-κB protein essential to the regulation of innate immunity *in vitro*

**Figure 6:**
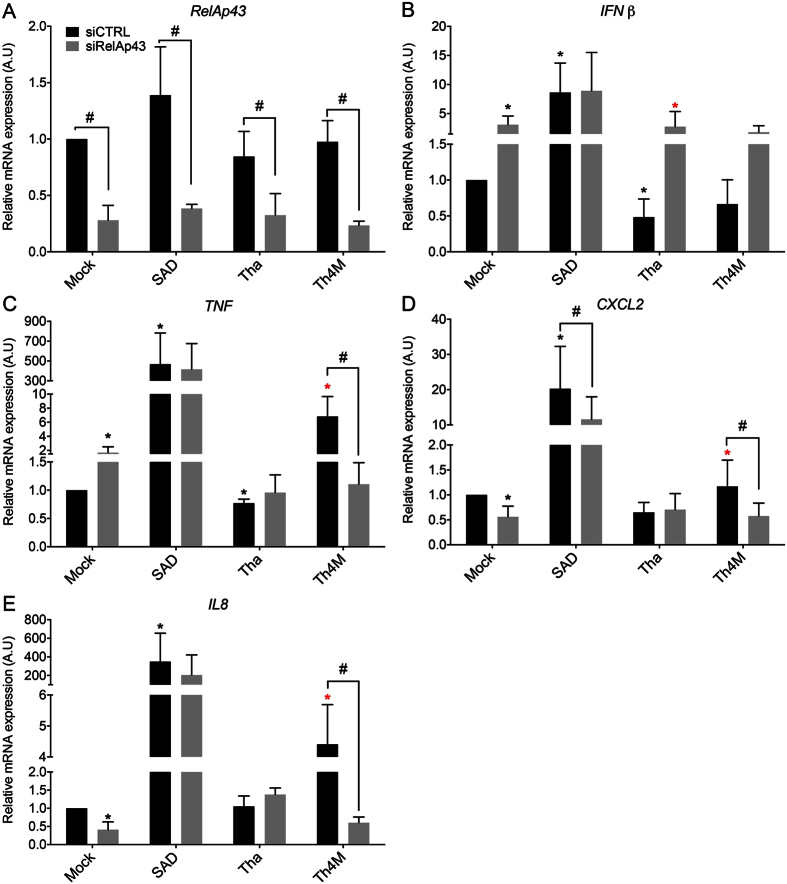
RelAp43 is involved in the modulation of genes related to innate immunity. HeLa cells were infected by Tha, Th4M or SAD virus and treated with a siRNA control (siCTRL) or a siRNA directed against RelAp43 (siRelAp43). After 48 hours of infection, total RNA was extracted. The efficiency of siRNA treatment was evaluated by the measure of *RelAp43* mRNA expression by RT-qPCR (**A**). The expression level of the following genes: *IFNβ* (**B**)*, TNF* (**C**)*, IL8* (**D**)*, CXCL2* (**E**) was studied by RT-qPCR analysis. Significant results (p < 0.05) compared to the siCTRL of mock (*) and Tha () infected cells are annoted. (^#^) indicated significant results between the siCTRL treated sample and the siRelAp43 respective one (p < 0.05). The levels of gene expression were normalized according to the level of *GAPDH* reporter gene in non-infected and untreated cells.

To confirm the key role of RelAp43 in the control of the host cell response to Tha infection, we assessed the effect of RelAp43 depletion on the selected genes. Their level of expression was quantified by real-time PCR in infected HeLa cells in which RelAp43 expression was depleted by specific siRNA in comparison to a siRNA control (Fig. 6A). The downregulation of *IFNß* and *TNF* as well as the control of the induction of *CXCL2* and *IL8* by Tha virus observed in Fig. 5 was here confirmed.

As expected, siRNA directed specifically against *RelAp43* gene induced a significant decrease of its transcription (more than 70%) in Tha, Th4M, SAD or mock infected Hela cells (Fig. 6A). As a consequence of *RelAp43* silencing, non infected HeLa cells overexpressed significantly (p < 0.05) *IFNβ* and *TNF* genes (Fig. 6B and C). Conversely, the transcription of *CXCL2* and *IL8* was significantly (p < 0.05) down regulated under depletion of RelAp43 (Fig. 6D,E). These results demonstrate that the physiologic level of RelAp43 expression in non infected Hela cells is characterized on the one hand by a higher level of transcription of *CXCL2* and *IL8* and on the other hand by a lower level of *TNF,* and *IFNβ* than in cells depleted in RelAp43, confirming the role of RelAp43 in the modulation of innate immune response.

Under SAD virus infection, depletion of RelAp43 induced no significant change in transcription of any of these genes but *CXCL2* (Fig. 6D), probably because of the strong activation of pathways by SAD that may bypass RelAp43. However, in the case of Tha virus, the depletion of RelAp43 increased significantly (p < 0.05) the transcription of *IFNβ* (Fig. 6B) to the level observed with the mock control. Hence, Tha virus is able to lower the expression of *IFNß* in presence of RelAp43 but losses this capacity when the later is depleted (Fig. 6B), confirming that Tha hijacks RelAp43 signaling. Further, the transcription of *TNF, CXCL2* and *IL8* were not affected by the siRelAp43 during Tha infection conversely to the mock control (Fig. 6C–E). Conversely, Th4M infection induced a significant decrease (p < 0.05) in expression of these 3 genes during depletion of RelAp43. Therefore the increase of transcription of *CXCL2, IL8* and *TNF* observed in the case of Th4M infection appeared to be dependent of RelAp43 and of the 4 positions mutated in Th4M.

### Matrix protein of Tha virus modulates the transcription of *TNF in vivo*

**Figure 7:**
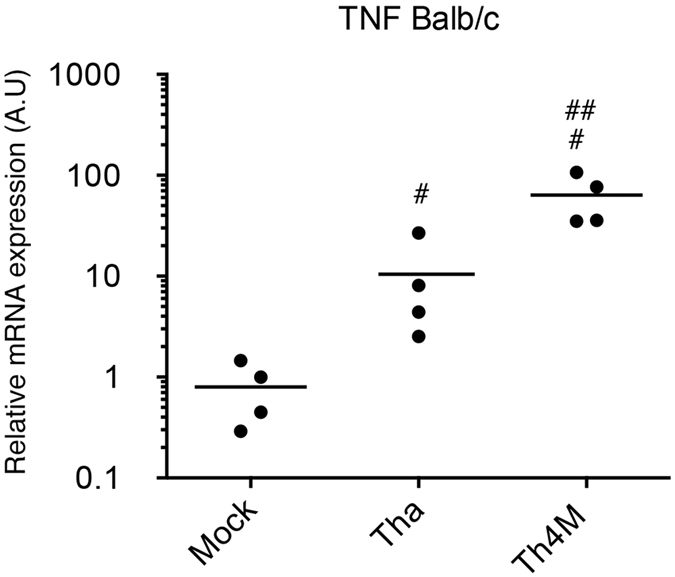
Matrix protein of Tha virus modulates the transcription of TNF *in vivo.* Relative quantification of *TNF* mRNA expression was performed in the brain of mice infected with Tha and Th4M viruses. Four six-weeks-old BALB/c were infected in intramuscular injection of 1000 FFU and monitored over 21 days. The mice were sacrified upon the apparition of late infection symptoms (humane endpoint) and RNA were extracted from the brain. The expression of the *TNF* mRNA was studied by RT-qPCR analysis. The levels of gene expression were normalized according to the level of *GAPDH* reporter gene in non-infected mice. Significant results (p < 0.05) compared to uninfected mice (^#^) and Tha (^##^) infected mice are annoted.

To confirme the modulation of RelAp43 pathway by the matrix protein of Tha virus, we quantified the transcription of *TNF* in the brain of mice during the ultimate phase of infection (at the humane endpoint) with Tha and Th4M viruses (Fig. 7). Each condition was tested on four mice. As expected, the transcription of *TNF* was significantly (p < 0.05) upregulated in Tha- or th4M-infected mice compared to non infected mice. However, the level of transcription of *TNF* gene induced by Th4M virus is significantly (p < 0.05) higher than that obtained with Tha virus. We confirm here that the matrix protein of Tha is able to hijack RelAp43 signaling, as it was shown before *in vitro*, therefore limiting the inflammatory response of the host.

## Discussion

The homeostasis of living cells is tightly regulated by transcription factors involved in signaling pathways. Among them, the NF-κB family regulates numerous genes controlling immune response, cell survival and proliferation together with tissue differentiation. It is therefore a target of choice for viral interference.

The data presented here confirmed that the M protein, one of the five proteins of lyssaviruses, the agents of rabies, is a potent regulator of the NF-κB signaling. This property, shared by 7 species of lyssavirus is in contrast to vaccine strains PV and SAD, and is mediated by the interaction of M protein with the C-terminal specific region of RelAp43, one of the six members of the NF-κB family^1^. The region of M protein, that RelAp43 binds to, comprises a sequence of 43 amino acids located in the central part of the M protein (amino acids in position 67 to 110) encompassing two α helices and part of a β strand^24^. In this short sequence, only 4 differences could account for the opposite properties of M proteins of a dog rabies virus isolate from Thailand (Tha) and vaccine strains. This was clearly shown by comparing the properties and effects of wildtype and different mutated M-Tha proteins. This was also completed by experiments in cell culture comparing Tha virus with its isogenic counterpart mutated in 4 different positions.

**Figure 8:**
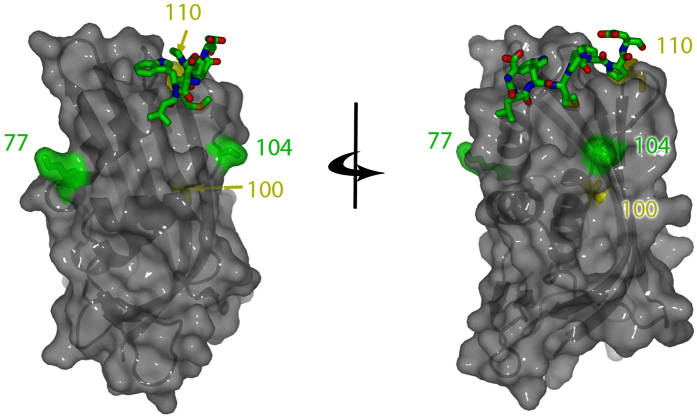
Location of residues of the M protein involved in the interaction with RelAp43. The M protein structure presented is that of Lagos bat virus^24^. Residues 77 and 104, the crucial residues for interaction with RelAp43 are colored in green. Positions 100 and 110 are in yellow. The residues from the N-terminal regions that interact with the globular domains are shown as sticks. The two views are rotated by 90 degrees.

Two conservative substitutions, R77K and A104S induced the loss of binding of M-Tha to RelAp43 but were not sufficient for restoring NF-κB-dependent reporter gene expression. To fully restore this effect, additional mutations, either D100A or M110L are required. Positions 77 and 104 although quite distant are located on the same side of the surface of the molecule which could explain their common important role (Fig. 8). The role of substitution D100A is certainly different. This position is internal and the non-conservative selected substitution would potentially disrupt the helix containing the amino acid in position 104. It is also interesting to mention that position 77 was described as a key residue for the activation of the extrinsic cell death pathway^28^. Thus, this region of the M protein seems to be crucial for the understanding of the modulation of host response after infection. The inhibitory effect of M from wild isolates of lyssavirus on NF-κB signaling is mediated by its action on RelAp43 where transcriptional modulation is probably strongly modified. This novel mechanism of transcriptional modulation of genes may be a common theme in coordinating activation of gene expression involved in innate immunity by viral proteins targeting NF-κB complex. Some viral strategies to counteract NF-κB response, particularly related to p65/RelA, have already been described. Positive strand RNA viruses like the *Picornaviridae* are known to degrade RelA and therefore suppress the innate immune response^30^,^31^. DNA viruses of the *Herpesviridae* family also prevent the transcriptional activity of NF-κB^32^. In measles virus, a *Paramyxoviridae*, more closely related to the *Rhabdoviridae*, the V protein produced from the P gene by a leaky scanning mechanism has been shown to specifically bind to the RHD of RelA and therefore to suppress NF-κB activity^33^. Thus, we expect that RelAp43, which shares with RelA the same N-terminal sequence including RHD, is likely to be targeted by other viruses than lyssavirus.

In the case of lyssavirus infection, this targeting of RelAp43 and the resulting inhibition of transcription of NF-κB-dependent genes is an additional interference against host response. Indeed, three other proteins of lyssavirus were already described as inhibitor of IRF3/7-dependant IFN induction and IFN signaling, the N, the P and the L protein^9^,^10^,^11^,^12^,^13^,^14^,^15^,^16^,^17^,^34^. Synergy between these four proteins in order to counteract the antiviral response could thus be important to understand lyssavirus pathogenicity.

The mechanism of transcriptional modulation mediated by RelAp43 remains an important issue. It is already known that RelAp43 can associate with the five other NF-κB proteins, in particular with p50 which is stabilized by RelAp43^1^. Doing so, RelAp43 can influence the active NF-κB dimer equilibrium and thus modulate the NF-κB response^4^. Since RelAp43 lacks the TAD, the intrinsic transactivation ability of the RelAp43-containing dimers has to be supported by the other NF-κB protein, either p65/RelA, RelB or c-Rel^5^. It is also possible that RelAp43 acts on gene expression by inducing the recruitment of secondary transcription factors on gene promoter, as described for p65/RelA^35^. Indeed, it was demonstrated that p65/RelA could still recruit some transcription factors when its TAD was deleted^35^. As RelAp43 is exactly similar to p65/RelA on its N-terminal part but lacks the TAD^1^, it probably also retains the ability to induce transcription via this TAD-independent mechanism. We cannot exclude that RelAp43 could also directly repress NF-κB target gene expression by κB site occupancy as a heterodimer with p50 (both lacking the TAD) and therefore enable the transactivation by p65/RelA. Indeed, we previously showed that RelAp43 is able to modify the equilibrium of p50-comprising dimers limiting the number of p65/RelA-p50 dimers^1^. In our study, we demonstrate a new role of RelAp43 in the modulation of cellular innate immune response.

RelAp43 is shown here to be a modulator of the expression of genes related to several cellular functions such as the IFN response (*IFNβ)* and inflammatory response (*TNF, CXCL2*, and *IL8*). Therefore, RelAp43 appears as a significant signaling protein potentially involved in the innate immune response against pathogens in general and a potential target for viral escape. Indeed, Tha virus hijack RelAp43 signaling to control the induction of the IFN and TNF and that was confirmed *in vivo* for *TNF*. All evidences here support that the expression of the inflammatory cytokines *TNF, IL8* and *CXCL2* is shown to be modulated by the interaction of the matrix protein of Tha virus with RelAp43. As such, rabies virus (Tha) and lyssaviruses^1^ in general may have developed specific strategies to inhibit host cellular response by interaction of the matrix protein with RelAp43 which is lost in rabies vaccine SAD strain but also by another mechanism independent of the matrix protein. Thus, RABV-M protein appears as a potent viral immune-modulatory factor that control NF-κB dependent genes expression by fixation of RelAp43.

## Materials and Methods

### Cell culture, transfections and infections

Human carcinoma epithelial (HeLa) and human epithelial kidney (HEK-293T) cells were grown using standard procedures in 37 °C humidified incubator with 5% CO_2_. All cell lines were grown in Dulbecco’s minimal essential medium (Gibco) supplemented with 10% heat-inactivated fetal bovine serum (Eurobio).

Transfection was performed using Lipofectamine 2000 (Invitrogen) as indicated by the provider. For siRNA transfection, DharmaFECT1 (Thermo Scientific) was used according to the manufacturer’s instructions. The siRNA sequences used were:

RelAp43 siRNA: AGGAGCAGUGGAGAUGAAGACUCUUGG

Control siRNA: CCCACCGAUGGAGGACUUUCAAUUU

For viral infection, rabies virus (RABV) from Thailand (referred as Tha, isolate 8743 THA) and a vaccine strain SAD, that were previously described^36^, were used. Cells were infected at a multiplicity of infection (m.o.i) of 1. To turn down endogenous RelAp43 expression in infected HeLa cells, control or RelAp43 siRNA were transfected as described above, 3 hours post infection.

When needed, 24 hours after transfection or infection, recombinant human TNF-α (R&D systems) was added (final concentration = 10 ng/ml) and incubated for indicated time at 37 °C.

### Construction of the reverse genetic system

Genes coding for N, P, and L proteins were inserted in the vector pTIT, which comprises the internal ribosome entry site (IRES) of the encephalomyocarditis virus (organization, T7 promoter-IRES-multiple cloning site-T7 terminator^37^ at NcoI and EcoRV restriction site. The N, P and L sequences were amplified by reverse transcription-PCR on viral RNA with specific primers (Table S2) allowing InFusion cloning.

The complete cDNA of Tha genome was constructed by replacing the sequence of SAD in pSDI-HH-flash-SC^38^ by the Tha sequence, between the *Pme*I and *RsrII* restriction sites. First, a synthetic gene encoding the leader end, the N initiation signal, a *SnaBI* restriction site, the non-coding region of the L and the trailer end of the genome was synthesized (Eurofin, MWG Operon, Germany). This synthetic gene was cloned using the InFusion technology (Clontech) in the pSDI-HH-flash-SC vector previously linearized in *PmeI* and *RsrII*. Then, the Tha sequence was divided in seven contiguous fragments using unique restrictions sites of the Tha genome (Supplementary Figure S2). For this, a linker was synthesized (Eurofin, MWG Operon, germany), encoding all the restriction sites needed for the several steps of construction and cloned at *SnaBI* restriction site. Then, 7 inserts were amplified by reverse transcription-PCR on viral RNA using the primers indicated in Supplementary Table S3 and cloned in the vector linearized with the restriction sites of the linker, using InFusion technology (Clontech). Sanger sequencing was used to control all clonings. The vector coding for the complete genome of THA was named pTharec.

The pTh4M plasmid was obtained by substitution of the fragment F3-1 of pTharec (Supplementary Figure S2) by mutation on residues 77, 100 104 and 110 of the M protein.

### Reverse genetic

Rescue of the recombinant virus named Th4M (mutated on residues 77, 100, 104 and 110 of the M protein) was performed by transfection full-length viral cDNA (2.5 μg) and plasmids N-pTIT (2.5 μg), P-pTIT (1.25 μg) and L-pTIT (1.25 μg) in 10^6^ BSR T7/5 cells^37^ grown in 6-well plates. After 3 days, transfected cells were passaged and incubated every three days. When 100% of the cells were infected, the supernatant was harvested and titrated on BSR cells. The infection was controlled by immunofluorescence using the FITC-conjugated anti-rabies virus nucleocapsid antibodies (Biorad).

### Recombinant plasmid construction and site-directed mutagenesis

Two RABV were used to generate clones coding for the M gene, as described previously^28^: Tha virus and vaccine strain SAD.

Some of the M-Tha deletion mutants described previously were used^26^ in this study after insertion in a pCI-neo-3xFLAG plasmid using the Gateway technology (Invitrogen). The deletion mutants M_46–202_ Tha and M_67–202_ Tha were designed following the same protocol using the primers shown in Table S2.

Point mutations in wild type M-Tha or M-SAD were performed using the QuickChange II site-directed mutagenesis kit (Stratagen) or Change IT multiple mutation site directed mutagenesis kit (USB) respectively, according to the manufacturer’s instructions. Primers used are shown in Table S3.

### Co-immunoprecipitation

Twenty-four hours post transfection, HeLa cells were harvested and proteins were extracted in an extraction buffer (300 mM NaCl, 50 mM Tris-HCL [pH8], 0.5% triton, 1 mM EDTA, protease inhibitor (Roche)) for 30 min at 4 °C, followed by centrifugation. Extracts containing 100 μg of total proteins were immunoprecipitated with the anti-FLAG M2 beads (Sigma, A8592) overnight at 4 °C or with a GFP-Trap-A (Chromotek, gta20) 1 hour at 4 °C. After centrifugation, beads were washed 3 times with washing buffer (500 mM NaCl, 50 mM Tris-HCL [pH8], 1 mM EDTA, protease inhibitor (Roche)). Proteins from the beads and input (15 μg) were separated by sodium dodecyl sulfate-polyacrylamide gel electrophoresis (SDS-PAGE) and analyzed in western blot experiments.

### Western blot analysis

Transfected cells were collected 24 hours post transfection, and proteins extracts were subjected to electrophoresis, using NuPAGE gels (Invitrogen), before transfer onto nitrocellulose membrane using iBlot transfer system (Invitrogen). Membranes were saturated for 30 minutes in PBS-Tween 0.1% with 5% non-fat dried milk and incubated with indicated antibodies. Proteins bands were revealed by chemiluminescence using the Amersham ECL kit.

### Antibodies

The following antibodies were used: mouse α-V5 antibody (Invitrogen); mouse α-FLAG M2 antibody and rabbit α-FLAG antibody (Sigma); mouse α-matrix 186-20 antibody^39^; mouse α-phosphoprotein 49-11 antibody^39^; mouse HRP-linked α-V5 antibody (Invitrogen) and mouse HRP-linked α-FLAG antibody (Sigma); mouse α-GFP antibody (JL8, Clontech); mouse β-Actin antibody (Sigma, A2228) and mouse secondary antibody coupled to peroxidase (GE Healthcare, NA931).

### Luciferase reporter gene assays

HEK-293T cells were plated in 96-well plates with a density of 20,000 cells per well in 100 μl of culture medium. After 24 h, cells were transfected with Lipofectamine 2000 (Invitrogen), as recommended by the provider. To measure the NF-κB response, we transfected cells with a mix containing (per well) FLAG-tagged M protein or CAT encoding plasmid (160 ng), pNF-κB-Luc (10 ng, Agilent Technologies) coding for firefly luciferase under control of κB sites, and EF1-β-gal (2 ng, gift from S. Memet, Institut Pasteur) encoding β-galactosidase under control of EF1 promoter insensitive to NF-κB activation. After 24 h post transfection and 5 hours of TNF treatment (final concentration of 10 ng/ml), luciferase and galactosidase activities were measured using Steady-glo and Beta-glo assays (Promega) respectively. Showed results represent the mean of three measures of each activity per transfection and are expressed as the luciferase/beta-galactosidase activity ratio.

### *In vivo* experiments

Six-weeks old BALB/c were infected by intramuscular injection of 1000 FFU and monitored over 21 days. Mice were sacrificed upon the apparition of late infection symptoms. The infection was confirmed by RT-qPCR.

The protocol of animal experiment was approved by the French Administration (Ministère de l’Enseignement et de la Recherche) under the number 2013-063 and all experiments were performed in accordance with the relevant guidelines and regulations. All animals were handled in strict accordance with good animal practice.

### RNA isolation and quantitative RT-PCR

Total RNA was extracted using RNAEasy Mini Kit (Qiagen) and reverse transcribed with Superscript II (Invitrogen) according to the manufacturer’s instructions. From the resulting synthesized single-stranded cDNA, 1/100 was used for each Real-time PCR in the presence of specific primers (1 mM) and Syber Green master mix (Applied Biosystems). Quantitative PCR were performed with a 7500 instrument (Stratagene). Each cDNA was normalized to histone deacetylase 1 (HDAC1), glyceralde-hyde-3-phosphate dehydrogenase (GADPH), and 18S ribosomal RNA. PCR were performed in duplicate and fitted to standard curves, providing mean cycle threshold values that were translated into arbitrary units corresponding to mRNA levels. Data were analyzed with 7500 SDS software version 2 (Applied Biosystems).

### Real-time PCR arrays

Quantitative mRNA expression analysis of genes was performed with the human NF-κB signaling target RT^2^ profiler PCR array (SABiosciences, QIAGEN). Total RNA was extracted from HeLa cells using RNAEasy Mini Kit (Qiagen). Reverse transcription was performed on total RNA (1 μg) using RT^2^ First strand kit following the manufacturer’s instruction, then real-time PCR array was performed in a 7500 instrument (Applied Biosystems) with the RT^2^ Real-Time SYBR Green PCR Master Mix (SABiosciences, QIAGEN) according to the manufacturer’s instruction.

### Statistics and densitometry analysis

Single comparisons of data were performed by Student’s *t* tests using the GraphPad Prism software. *p* values under 0.05 were considered significant.

## Additional Information

**How to cite this article**: Ben Khalifa, Y. *et al*. The matrix protein of rabies virus binds to RelAp43 to modulate NF-κB-dependent gene expression related to innate immunity. *Sci. Rep.* **6**, 39420; doi: 10.1038/srep39420 (2016).

**Publisher's note:** Springer Nature remains neutral with regard to jurisdictional claims in published maps and institutional affiliations.

## Supplementary Material

Supplementary Data
