# Supplementary material for: The matrix protein of rabies virus binds to RelAp43 to modulate NF-κB-dependent gene expression related to innate immunity
Source: Sci Rep. 2016 Dec 21;6:39420. doi: 10.1038/srep39420 (PMC5175135; doi:10.1038/srep39420)
Supplement: Supplementary Data [file srep39420-s1.pdf]

SUPPLEMENTAL DATA

The matrix protein of rabies virus binds to RelAp43 to modulate NF-κB-dependent gene expression related to innate immunity

Youcef Ben Khalifa [(1)#], Sophie Luco [(1, 2) #], Benoit Besson[(1, 2)], Florian Sonthonnax[(1, 2)], Medhi Archambaud[(1, 2)], Jonathan M. Grimes [(3,4)], Florence Larrous[(1)] and Hervé Bourhy *[(1)]

Table S1: Tha infection induces a strong repression of the NF-κB signaling target genes compared to SAD infection. Genes found significantly ($p<0.05$) modulated in the NF-κB signaling PCR array data are listed by alphabetical order. Values given corresponds to the fold up- down- regulation factors in Tha infected cells compared to SAD infected cells. The complete list of genes can be found at the website of the manufacturer. (http://www.sabiosciences.com/rt_pcr_product/HTML/PAHS-225Z.html).

p-value	Fold Change	Symbol	Description
0,0144	-1,77	BIRC2	**Baculoviral IAP repeat containing 2**
0,0145	-4,12	BIRC3	**Baculoviral IAP repeat containing 3**
0,0041	-3,22	CCL2	**Chemokine (C-C motif) ligand 2**
0,0202	-11,01	CCL5	**Chemokine (C-C motif) ligand 5**
0,0105	-1,62	CCR5	**Chemokine (C-C motif) receptor 5**
0,0687	-3,40	CSF1	**Colony stimulating factor 1 (macrophage)**
0,0064	-40,48	CXCL2	**Chemokine (C-X-C motif) ligand 2**
0,0022	-5,73	CXCL9	**Chemokine (C-X-C motif) ligand 9**
0,0184	-3,43	IFNB1	**Interferon, beta 1, fibroblast**
0,0126	-5,09	IFNG	**Interferon, gamma**
0,0445	-30,86	IL8	**Interleukin 8**
0,0077	-42,72	INS	**Insulin**
0,0151	-3,71	TNFB	**Lymphotoxin alpha (TNF superfamily, member 1)**
0,0288	-27,39	MAP2K6	**Mitogen-activated protein kinase kinase 6**
0,0001	2,15	MITF	**Microphthalmia-associated transcription factor**
0,0212	-2,54	NFKB2	**Nuclear factor of kappa light polypeptide gene enhancer in B-cells 2 (p49/p100)**
0,0079	-4,36	NFKBIA	**Nuclear factor of kappa light polypeptide gene enhancer in B-cells inhibitor, alpha**
0,0053	-1,71	NR4A2	**Nuclear receptor subfamily 4, group A, member 2**
0,0232	-4,14	RELB	**V-rel reticuloendotheliosis viral oncogene homolog B**
0,0187	-7,40	SOD2	**Superoxide dismutase 2, mitochondrial**
0,0090	-133,15	TNF	**Tumor necrosis factor**

Table S2: Primers used for cloning and mutagenesis.

The sequences of the different primers were directed from 5′ to 3′. For, forward; Rev, reverse

(*) Primers were phosphorylated in 5'.

**Construct**		**Primer sequence (5' to 3')**
M-SAD	For	ATGAACCTCCTACGTAAGATAGTGAAAAACCGC
	Rev	TTATTCTAGAAGCAGAGAGGAATC
$M_{67\text{-}202}$ Tha	For	GAGGAGATCTTACTCCTTCAAGATACTC
	Rev	TGGGAAGCTTGCTATTCTAGGAGCAGGGAAGAGTC
$M_{46\text{-}202}$ Tha	For	GAGGAGATCTGGCAAGGCCAGTGTGAGAAAC
	Rev	TGGGAAGCTTGCTATTCTAGGAGCAGGGAAGAGTC
M-Tha 77	For	GGATCCTGCGGCACATTCTAAAGTCATTCGATAATATATATTCTGG
	Rev	CCAGAATATATATTATCGAATGACTTTAGAATGTGCCGCAGGATCC
M-Tha 100	For	GGTAGTTATTGGACTGGCTTTATCAGGAGCTCCAG
	Rev	CTGGAGCTCCTGATAAAGCCAGTCCAATAACTACC
M-Tha 104	For	GTTATTGGACTGGATTTATCAGGAAGTCCAGCTCCTGAGGG
	Rev	CCCTCAGGGACTGGACTTCCTGATAAATCCAGTCCAATAAC
M-Tha 110	For	TCCAGTCCCTGAGGGCTTGAATTGGGTATACAAA
	Rev	TTTGTATACCCAATTCAAGCCCTCAGGGACTGGA
M-SAD 77	For (*)	GGCACATTCTAAGATCATTCGAC
M-SAD 100	For (*)	ATTGGACTGGATTTGTCAGGAT
M-SAD 104	For (*)	TTTGTCAGGAGCTCCAGTCCC
M-SAD 110	For (*)	CCCTGAGGGCATGAACTGGGT
Ntha-pTIT	For	GATGATAATACCATGGATGCCGACAAGATTG
	Rev	CTGCAGGAATTCGATTTACGAGTCACTTGAATATG
Ptha-pTIT	For	GATGATAATACCATGAGCAAGATCTTTGTCAAT
	Rev	CTGCAGGAATTCGATTCAGCGGGATGTATAACGG
Ltha-pTIT	For	GATGATAATACCATGTTGATCGATTCAGGAG
	Rev	CTGCAGGAATTCGATTCATAAGCAACTGTAGTCTAG

Table S3: Primers used for cloning the complete genome of Tha

The sequences of the different primers were directed from 5′ to 3′. For, forward; Rev, reverse. The length of each amplified fragment is indicated. (*) Fragment F2 is a linker harbouring all the restriction sites necessary for the construction of the plasmid.

Frag 1<br>1356 pb	For	ATGTAACACCCCTACAATGGATGCCGACAAG
	Rev	GAAATCTCCAGCTACGTATTTAGTCGACCTCCGTTCATC
Frag 2 (*)	For	CATGATGAACGGAGGTCGACTATACCCGGGTATAGCTAGCTATACTCGAGTATACGTAGCTGGAGATTTC
	Rev	GAAATCTCCAGCTACGTATACTCGAGTATAGCTAGCTATACCCGGGTATAGTCGACCTCCGTTCATTCATG
Frag 4<br>2579 pb	For	CTTGAGGAAGCTCGTCCCGGGTTTGGGAAG
	Rev	CTCGAGTATAGCTAGCCTCGTTCTGGTGAAGGAGTGTG
Frag 5<br>1393 pb	For	CTTCACCAGAACGAGGCTAGCGTCTTGGTTG
	Rev	GCTACGTATACTCGAGTGTTCTCTCTCCAAGATCTGG
Frag 6-1<br>2181 pb	For	AGAGAGAACACTCGAGAGCTTCAC
	Rev	TATATACGTAGGCGCCAGTTGCCCACTG
Frag 6-2<br>1507 pb	For	TATACTCGAGGGCGCCCATTATAAGC
	Rev	CTCTTACGTATCATAAGCAACTGTAGTCTAGC
Frag 3-1<br>1568 pb	For	ATGAACGGAGGTCGACTAAAGAG
	Rev	CCCCTCAAGGGGACCCCTAGAATCGGCCCTA
Frag 3-2<br>1418 pb	For	TATAGTCGACCTAGGGGTCCCCTTGAGG
	Rev	CTTTCCCAAACCCGGGGACGAGCTTCCTCAAGTGAC

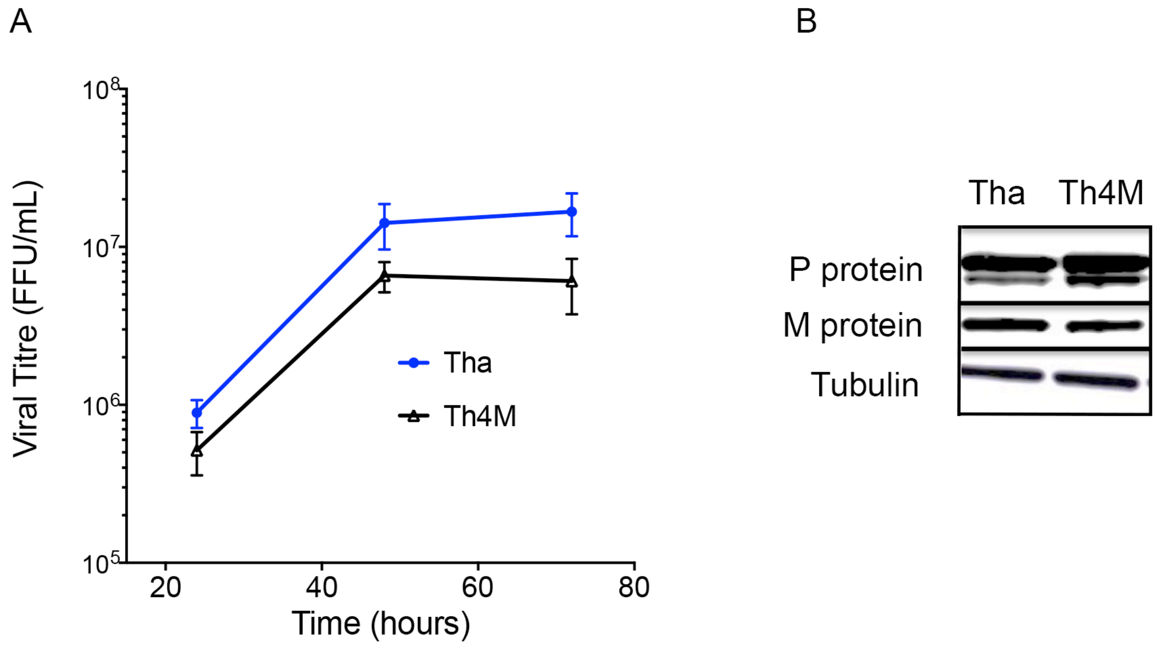

Figure S1: Rescue of recombinant Tha virus mutated on residues 77-100-104-110.

(A) Growth curves of the recombinant virus. The recombinant virus Th4M and the wild type virus Tha were inoculated on BSR cells at a MOI of 0.1 and supernatants were recovered at 24h, 48h and 72h p.i., and then titrated on BSR cells. The infectious titres are expressed in FFU/ml.

(B) Expression of viral proteins of Th4M virus. The expression of P and M proteins was controlled by western blot using monoclonal antibodies directed against the P protein (Ac 49-1 targeting several isoforms of P protein) or the M protein (Ac 186-20).

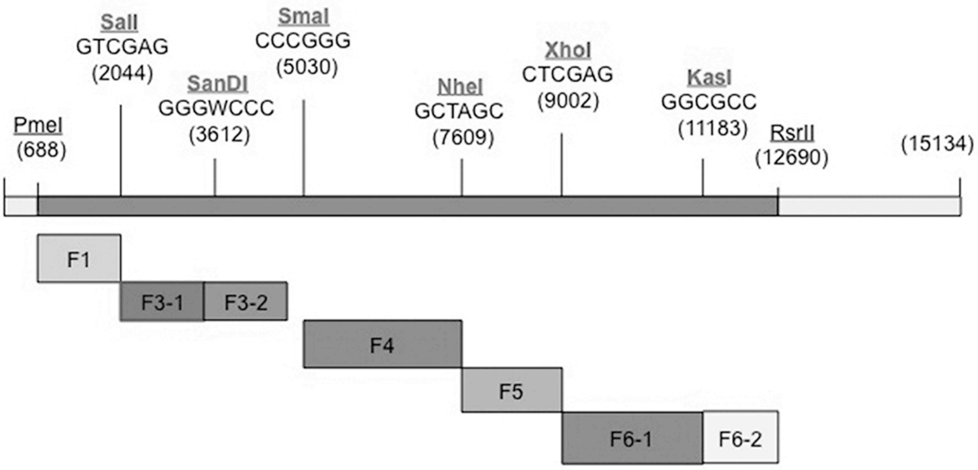

Figure S2: Construction of the complete cDNA coding for THA genome. The genome of THA was built using unique restriction sites present in the viral genome sequence. The genome was divided into five blocks shown in the diagram; two of them (F3 and F6) were further subdivided into two more parts to facilitate cloning. First, the fragment F1 was inserted in the pSDI-HH-flash-SC vector at *SnaBI* site and then we added fragment F2 harbouring all the restriction sites necessary for the construction of the complete genome of Tha. The fragments F4, F5; F6-1, F6-2, F3-2 and finally F3-1 were introduced one after the other. Mutations in the matrix gene were introduced in fragment F3-1. Positions of each fragment in the pTharec vector are indicated.
